# Coagulation and inflammation in scrub typhus and murine typhus—a prospective comparative study from Laos

**DOI:** 10.1111/j.1469-0691.2011.03717.x

**Published:** 2011-11-07

**Authors:** D H Paris, V Chansamouth, P Nawtaisong, E C Löwenberg, R Phetsouvanh, S D Blacksell, S J Lee, A M Dondorp, T van der Poll, P N Newton, M Levi, N PJ Day

**Affiliations:** 1 Mahidol-Oxford Research Unit, Faculty of Tropical Medicine, Mahidol UniversityBangkok, Thailand; 2 Centre for Tropical Medicine, Nuffield Department of Clinical Medicine, Churchill HospitalHeadington, Oxford, UK; 3 Wellcome Trust–Mahosot Hospital–Oxford Tropical Medicine Research Collaboration, Mahosot HospitalVientiane, Lao PDR; 4 Department of Vascular Medicine and Centre for Experimental and Molecular Medicine (CEMM), Academic Medical Centre, University of AmsterdamAmsterdam, The Netherlands

**Keywords:** Coagulation, inflammation, Laos, murine typhus, pathophysiology, scrub typhus, vasculitis

## Abstract

Scrub typhus (caused by *Orientia tsutsugamushi*) and murine typhus (caused by *Rickettsia typhi*) cause up to 28% of febrile episodes in Thailand and Laos. The current understanding of coagulation and inflammation in the pathogenesis of these clinically very similar vasculotropic diseases is limited. This study compared human *in vivo* changes in 15 coagulation, inflammation and endothelial activation markers in prospectively collected admission and follow-up samples of 121 patients (55 scrub typhus, 55 murine typhus, and 11 typhus-like illness) and 51 healthy controls from Laos. As compared with controls, all but one of the markers assessed were significantly affected in typhus patients; however, the activation patterns differed significantly between scrub and murine typhus patients. The levels of markers of coagulation activation and all inflammatory cytokines, except for interleukin-12, were significantly higher in patients with scrub typhus than in those with murine typhus. In patients with murine typhus, however, the levels of endothelium-derived markers were significantly higher. Anticoagulant factors were inhibited in both typhus patient groups. This is the first study demonstrating that, in scrub typhus, *in vivo* coagulation activation is prominent and is related to a strong proinflammatory response, whereas in murine typhus, changes in coagulant and fibrinolytic pathways are suggestive of endothelial cell perturbation. These data suggest that, although late-stage endothelial infection is common in both diseases, the *in vivo* pathogenic mechanisms of *R. typhi* and *O. tsutsugamushi* could differ in the early phase of infection and may contribute to disease differentiation.

## Introduction

Rickettsial diseases are important and under-recognized causes of morbidity and mortality in Southeast Asia. Whereas Rocky Mountain spotted fever (caused by *Rickettsia rickettsii*) and Mediterranean spotted fever (*Rickettsia conorii*) are prevalent in the western hemisphere and the Mediterranean basin, scrub typhus (*Orientia tsutsugamushi*) and murine typhus (*Rickettsia typhi*) are responsible for the majority of rickettsial diseases in Asia, and cause up to 28% of febrile episodes in Thailand and the Lao People’s Democratic Republic (Laos) [1,2]. An increasing proportion of returning travellers are at risk of acquiring scrub or murine typhus [3]. The true incidence of these diseases is likely to be underestimated, owing to suboptimal diagnostic tools [4] and the limited availability of epidemiological data. Patients with scrub or murine typhus commonly present with fever and non-specific symptoms, but both diseases can be complicated by meningoencephalitis, disseminated intravascular coagulation (DIC), or severe pneumonitis, which may culminate in acute respiratory distress syndrome [5].

The cellular tropism for spotted fever group and typhus group *Rickettsia* has been shown to be mainly endothelial [6,7], and observations in scrub typhus point to the endothelium as the main site of late-stage infection [8], but *in vivo* data are lacking. Recent findings of significantly raised *in vivo* levels of soluble L-selectins in scrub typhus patients [9] suggest mononuclear cell activation rather than endothelial activation at the hospital admission time-point, which may represent *O. tsutsugamushi* tropism during early dissemination, or local immune activation within the eschar and draining lymph nodes.

The current study was aimed at comparing *in vivo* levels of coagulation and inflammation markers in patients with acute murine typhus and acute scrub typhus in order to understand the roles of early vasculopathic changes accompanying these disease states.

## Materials and Methods

### Study population

A total of 248 non-pregnant patients with clinical suspicion of scrub typhus or murine typhus were prospectively recruited at Mahosot Hospital, Vientiane, Lao PDR. Of these, 121 patients with paired positive dynamic serology findings were randomly selected, including 55 patients with scrub typhus, 55 patients with murine typhus, 11 ‘febrile controls’ with clinical suspicion of typhus, but negative paired serology and PCR results for scrub and murine typhus, and 51 local contemporary blood donors as healthy controls.

### Ethics statement

The study was approved by the National Ethics Committee for Health Research, Ministry of Public Health, Lao PDR, and the Oxford Tropical Research Ethics Committee, UK. All patients gave written informed consent prior to sample collection.

### Investigations

On admission, a full physical examination and the following panel of investigations were performed: complete blood count, haematological and biochemical markers (Table 1), indirect immunofluorescence assays (IFAs), PCR assays, and coagulation (ELISA) and cytokine markers (flowcytometric assay (FACS)). All follow-up samples, which were available for all patients, were processed for IFA, coagulation and cytokine measurements.

**TABLE 1:** Demographic, clinical and laboratory characteristics of patients

Parameter	Unit	Scrub typhus	Murine typhus	p-value (ST vs. MT)	Febrile controls
Age	Years (range)	26 (5–75)	31 (9–82)	0.13	27 (16–65)
Days of fever^a^	Days (IQR)	8.5 (7–11)	8 (7–10)	0.54	8 (7–15)
ADM-FUP^b^	Days (IQR)	6 (4–7)	6 (4–7)	0.50	3 (3–6)
Eschar	No. (%)	23/54 (43)	0/55 (0)	**0.0001**	2/11 (18)
Skin rash	No. (%)	10/54 (19)	10/54 (19)	0.93	3/11 (27)
Lymphadenopathy^c^	No. (%)	34/54 (63)	3/54 (6)	**0.0001**	0 (0)
Haemorrhage^d^	No. (%)	22/55 (40)	5/55 (9)	**0.0002**	3/8 (38)
Hearing loss	No. (%)	24/30 (80)	0/15 (0)	0.07	1/8 (13)
GCS	Score (IQR)	15 (15–15)	15 (15–15)	0.32	15 (15–15)
WBC	×10^3^/mL (IQR)	9.6 (6.5–12.80)	8.5 (6.8–10.7)	0.49	7.3 (6.2–7.9)
Lymphocytes	% WBC (IQR)	30 (22–40)	33 (27–40)	0.34	36 (33–41)
Monocytes	% WBC (IQR)	4.5 (0–9)	1 (0–4)	0.62	0.5 (0–1)
Platelets	1000/mL (IQR)	209 (182–225)	200 (170–210)	0.11	210 (170–250)
Sodium	mmol/L (IQR)	137 (132–143)	145 (139–151)	**0.0003**	145 (141–148)
Creatinine	μmol/L (IQR)	88.4 (70.7–114.9)	106.1 (88.4–123.8)	**0.006**	106.1 (97.2–132.6)
Albumin	g/dL (IQR)	3.3 (2.7–3.7)	3.9 (3.3–4.2)	**0.0002**	4.2 (3.6–4.9)
Blood urea nitrogen	mmol/L (IQR)	3.93 (3.21–5.0)	3.2 (2.5–5.0)	0.11	3.57 (2.5–3.9)
Aspartate transaminase	U/L (IQR)	84 (52–130)	75 (44–102)	0.24	64 (22–89)
C-reactive protein	U/L (IQR)	81 (46–131)	48 (30–113)	**0.05**	2 (1–64)
Lactate dehydrogenase	U/L (IQR)	514 (389–626)	429 (324–555)	**0.03**	389 (274–546)

ADM-FUP, time between admission and follow-up; GCS, Glasgow Coma Scale; IQR, interquartile range; MT, murine typhus; ST, scrub typhus; WBC, white blood cell count.

Comparisons of demographic, clinical, haematological and biochemical parameters for scrub typhus (*n* = 55), murine typhus (*n* = 55) and febrile controls (*n* = 11). Significant p-values are depicted in bold. Probability values were calculated with the Kruskal–Wallis equality-of-populations rank test.

a Represents the number of febrile days before admission.

b The admission to follow-up period for cytokine, coagulation and biochemistry parameters (not identical to the period between paired diagnostic samples for serology).

c Regional and/or generalized lymphadenopathy.

d The criteria for ‘haemorrhage’ were defined as (muco)cutaneous petechial and suffusion bleeding sites.

### Serological diagnosis

The definitive diagnoses of scrub typhus and murine typhus were based on a ≥4-fold dynamic rise in IgM and IgG IFA titres for paired serum samples, which represents the current serological reference standard [4]. Slides prepared and standardized by the Australian Rickettsial Reference Laboratory were used for anti-*O. tsutsugamushi* antibody detection (using pooled Karp, Kato and Gilliam antigens) and anti-*R. typhi* antibody detection (*R. typhi* Wilmington strain antigens).

### Molecular diagnosis

On admission, bacteraemic patients were identified by real-time PCR, targeting the *groEL* gene for scrub typhus [10] and the *ompB* gene for murine typhus [11], as previously described, with modification of the endpoint visualization by intercalating SYBR green [12]. DNA templates were extracted from 200 μL of buffy coat collected from EDTA-anticoagulated full blood samples (Qiagen Mini Blood kit; Qiagen, Germantown, MD, USA).

### Cytokines

The plasma concentrations of inflammatory cytokines (Table 2) were measured by flow-cytometric bead assay according to the manufacturer’s instructions (Cat. No. 551811; BD Biosciences, San Jose, CA, USA). The detection limit for each analyte was determined by use of a serial dilution of the provided recombinant standard to generate a standard curve (curve-fitting model; four-parameter logistic): 2.9 pg/mL for interleukin (IL)-12, 4.8 pg/mL for tumour necrosis factor-α (TNF-α) and IL-1β, 5.4 pg/mL for IL-6, 4.5 pg/mL for IL-8, and 4.6 pg/mL for IL-10.

**TABLE 2:** Markers of coagulation and inflammation on admission in patients and controls

		Median (IQR)				p-values			
			
Parameter	Unit	ST	MT	FC	HC	ST vs. MT	ST vs. FC	MT vs. FC	Typhus vs. Controls
TAT complexes	ng/mL	18.2 (16.9–19.3)	11.6 (10.6–12.9)	6.8 (5.7–8.2)	3.8 (3.0–4.8)	**0.0001**	**0.0001**	**0.0001**	**0.0001**
sTF	pg/mL	538 (467–587)	298 (276–326)	87 (72–98)	43 (32–59)	**0.0001**	**0.0001**	**0.0001**	**0.0001**
sTM	ng/mL	228 (203–254)	498 (456–537)	113 (105–123)	91 (84–102)	**0.0001**	**0.0001**	**0.0001**	**0.0001**
VWF	%	176 (143–201)	302 (276–361)	112 (104–125)	103 (89–111)	**0.0001**	**0.0004**	**0.0001**	**0.0001**
AT	%	89 (84–95)	104 (98–110)	101 (98–103)	103 (98–108)	**0.0001**	**0.0013**	0.255	**0.0007**
PC	%	99 (93–105)	82 (75–88)	102 (98–108)	102 (94–107)	**0.0001**	0.3053	**0.0001**	**0.0001**
PAI-1	ng/mL	12.8 (11.5–16.6)	20.5 (16.8–23.8)	7.8 (6.4–9.1)	5.2 (3.7–6.8)	**0.0001**	**0.0001**	**0.0001**	**0.0001**
PAA	%	92 (89–94)	84 (80–88)	103 (102–107)	102 (94–106)	**0.0001**	**0.0001**	**0.0001**	**0.0001**
tPA	ng/mL	11.4 (8.6–13.7)	16.4 (14.8–18.9)	7.1 (5.9–8.2)	4.7 (3.7–5.9)	**0.0001**	**0.0008**	**0.0001**	**0.0008**
TNF-α	pg/mL	1.4 (1.1–2.4)	0 (0–1.3)	1.3 (0–2.7)	0 (0–1.1)	**0.0003**	0.985	0.069	**0.01**
IL-1β	pg/mL	0 (0–3.5)	0 (0–0)	0 (0–2.8)	0 (0–0)	**0.0002**	0.931	**0.005**	0.16
IL-6	pg/mL	17.7 (12.1–37.2)	12.7 (7.7–23.3)	7.9 (2.2–20.8)	0 (0–1.3)	**0.02**	0.178	**0.02**	**0.0001**
IL-8	pg/mL	17.5 (12.6–26.1)	11.5 (8.0–24.6)	13.1 (4.3–27.4)	4.6 (3.1–6.9)	**0.04**	0.167	0.654	**0.0001**
IL-10	pg/mL	13.5 (6.4–25.9)	3.0 (1.6–4.8)	4.1 (0–13.4)	0 (0–0)	**0.0001**	**0.036**	0.69	**0.0001**
IL-12	pg/mL	1.7 (0–2.4)	1.6 (0–2.2)	1.3 (0–1.7)	1.3 (0–1.9)	0.42	0.39	0.59	**0.03**

AC, all controls; AT, antithrombin; FC, febrile controls; HC, healthy controls; IL, interleukin; IQR, interquartile range; MT, murine typhus group; PAA, plasminogen activator activity; PAI-1, plasminogen activator inhibitor-1; PC, protein C; ST, scrub typhus group; sTF, soluble tissue factor; sTM, soluble thrombomodulin; TAT, thrombin–antithrombin; TNF-α, tumour necrosis factor-α; tPA, tissue-type plasminogen activator; VWF, von Willebrand factor.

Results of admission samples only. Probability values were calculated with the Kruskal–Wallis equality-of-populations rank test. Sample sizes for coagulation parameters: ST, *n* = 55; MT, *n* = 55; FC, *n* = 11; HC, *n* = 51. Sample sizes for cytokines: ST, *n* = 51; MT, *n* = 49; FC, *n* = 11; HC, *n* = 45.

Significant p-values are depicted in bold.

### Markers of coagulation, fibrinolysis, and endothelium activation

Thrombin–antithrombin (TAT) complexes, soluble tissue factor (sTF), plasminogen activator inhibitor-1 (PAI-1), tissue-type plasminogen activator (tPA), plasminogen activator activity (PAA), antithrombin (AT), protein C (PC), soluble thrombomodulin (sTM) and von Willebrand factor (VWF) were measured in all samples. TAT complexes, sTF, tPA and sTM concentrations were measured with commercially available ELISAs (TAT complexes, Behringwerke AG, Marburg, Germany; sTF, American Diagnostics, Greenwich, CT, USA; tPA and sTM, Diagnostica Stago, Asnières-sur-Seine, France). AT, PAA and PAI-1 activities were measured with automated amidolytic techniques. PC activity was determined with an amidolytic assay using chromogenic substrate S2366 (Chromogenix, Milan, Italy). VWF antigen was assessed by ELISA with commercial antibodies (Dako, Glastrup, Denmark), as described previously [13].

### Statistical analysis

Results are reported as medians and interquartile ranges, unless otherwise specified. Patient groups were compared by use of the Kruskal–Wallis test. All statistical analyses, including receiver operating characteristic curves of coagulation parameters, were calculated with Stata/MP 11.0 (Stata Corp., College Station, TX, USA).

## Results

### Demographic, clinical and laboratory characteristics of patients

The median intervals (interquartile range) between admission and convalescent serology were 7 days (5–12 days) for scrub typhus patients, 8 days (5–14 days) for murine typhus patients, and 6 days (4–13 days) for febrile controls (p 0.7) (Table 1). The proportions of patients with bacteraemia were 47/55 (85%) in scrub typhus patients and 43/55 (78%) in murine typhus patients. All typhus patients survived to discharge, had similar histories of ‘duration of fever’ prior to admission, and had similar demographic and haematological parameters. Laboratory parameters that differed between the two typhus groups were plasma sodium, creatinine, albumin, C-reactive protein (CRP) and lactate dehydrogenase (Table 1), although sodium and creatinine values remained within the normal range for both groups. On the basis of these data, the disease severity did not appear to differ significantly between the two patient groups, allowing for direct comparisons.

Upon admission, three clinical features were significantly associated with scrub typhus: the presence of eschar (p <0.001), lymphadenopathy (p <0.001), and mucosal/skin haemorrhages (p <0.001). Hearing loss was only observed in patients with scrub typhus (p 0.07), whereas skin rash was observed in equal proportions in both forms of typhus.

### Markers of coagulation, fibrinolysis, and endothelium activation

**FIG. 1:**
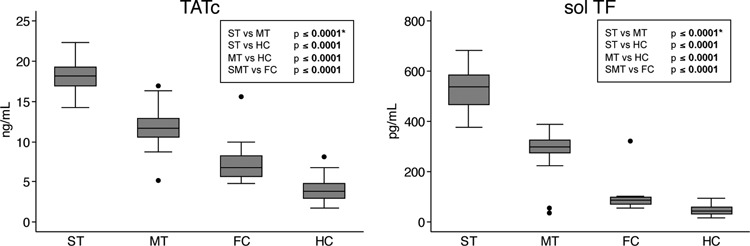
Markers of coagulation. Levels of thrombin–antithrombin (TAT) complexes and soluble tissue factor (sTF) were significantly higher in patients with scrub typhus (ST), murine typhus (MT) and scrub and murine typhus combined (SMT) than in febrile controls (FC) or healthy controls (HC). On comparison of ST and MT, TAT complexes and sTF levels were significantly higher in ST. Data are expressed as mean and interquartile range (grey boxes) and upper and lower adjacent values (whiskers) of admission samples. *Odds ratios (95% CIs): TAT complexes, 0.24 (0.13–0.44); sTF, 0.89 (0.78–1.01).

The plasma levels of all markers were significantly different in patients with scrub or murine typhus than in controls, except for AT in patients with murine typhus and PC in patients with scrub typhus. However, the activation patterns differed significantly between the two patient groups (Table 2). Coagulation activation, with high plasma concentrations of TAT complexes and sTF, was more pronounced in patients with scrub typhus than in patients with murine typhus (p <0.001) (Fig. 1).

**FIG. 2:**
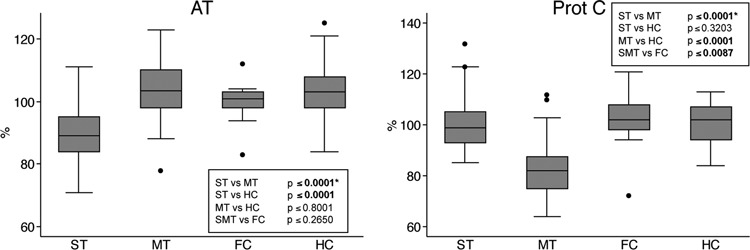
Anticoagulant factors. On admission, anticoagulant pathways were inhibited in patients with scrub typhus (ST), murine typhus (MT) and scrub and murine typhus combined (SMT) as compared with controls. Antithrombin (AT) levels were significantly decreased in ST but not in MT, whereas protein C (PC) levels were significantly lower in MT, but not in ST, than in healthy controls (HC). Data are depicted as mean and interquartile range (grey boxes) and upper and lower adjacent values (whiskers). *Odds ratios (95% CI): AT, 1.17 (1.1–1.24); PC, 0.80 (0.73–0.87). FC, febrile controls.

Overall anticoagulant activity was decreased in both typhus groups: in patients with scrub typhus, but not in those with murine typhus, AT levels were significantly decreased (p <0.001, p 0.8), whereas PC levels were significantly lower in patients with murine typhus, but not in those with scrub typhus (p <0.001 and p 0.3) (Fig. 2).

**FIG. 3:**
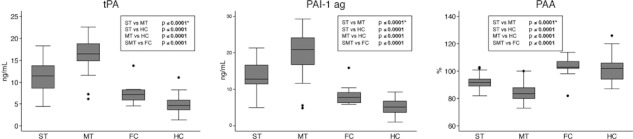
Markers of fibrinolytic activity. Although, on admission, levels of tissue-type plasminogen activator (tPA) and plasminogen activator inhibitor-1 (PAI-1) were significantly raised, overall plasminogen activator activity (PAA) was significantly lower in patients with murine typhus (MT) than in those with scrub typhus (ST). Data are expressed as mean and interquartile range (grey boxes) and upper and lower adjacent values (whiskers). *Odds ratios (95% CIs): tPA, 1.69 (1.39–2.05); PAI-1, 1.39 (1.23–1.57); PAA, 0.78 (0.71–0.86). FC, febrile controls; HC, healthy controls; SMT, scrub and murine typhus combined.

**FIG. 4:**
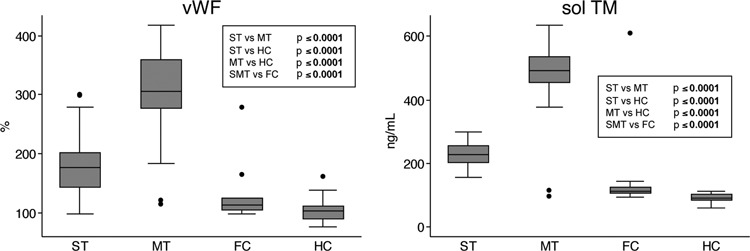
Markers of endothelial cell perturbation. On admission, plasma concentrations of endothelium-derived soluble thrombomodulin (sTM) and von Willebrand factor (VWF) were significantly higher in patients with murine typhus (MT) than in those with scrub typhus (ST). Data are depicted as mean and interquartile range (grey boxes) and upper and lower adjacent values (whiskers). *Odds ratios (95% CIs): VWF and sTM, 1.03 (1.02–1.04). FC, febrile controls; HC, healthy controls; SMT, scrub and murine typhus combined.

Levels of endothelial cell-derived tPA were higher in patients with murine typhus than in those with scrub typhus. PAA levels were decreased in both typhus groups as compared with healthy controls, but the decrease was more pronounced in patients with murine typhus, corresponding with increased PAI-1 levels in these patients (Fig. 3). Plasma concentrations of sTM and VWF were elevated in both typhus groups as compared with controls, but the increases were significantly more prominent in patients with murine typhus (p <0.001 for both indices) (Fig. 4).

### Markers of inflammation

In patients with scrub typhus, the levels of all cytokines measured were significantly raised as compared with healthy controls, whereas in patients with murine typhus, the levels of all except IL-1β were raised. Plasma levels of IL-6, IL-8 and, particularly IL-10, were markedly higher in patients with scrub typhus than in those with murine typhus (Table 2). On admission, TNF-α and IL-1β levels were low in both typhus patient groups: 39/51 (76%) and 18/51 (35%) of patients with scrub typhus had detectable levels of TNF-α and IL-1β, respectively, and 23/49 (47%) and 3/49 (6%) of patients with murine typhus had detectable levels of TNF-α and IL-1β, respectively. Because of an expected proportion of TNF-α and IL-1β levels being under the limit of detection, the resulting medians for both groups could be 0, although the proportions of detectable levels differed sufficiently to allow the generation of a statistically significant p-value (both p ≤0.001).

### Power of prediction/discrimination

All coagulation markers were further evaluated for their diagnostic potential and ability to correctly classify patients with either form of typhus (healthy controls were excluded). Only TAT complexes, sTF, VWF, sTM and tPA demonstrated sufficient discrimination, with areas under the receiver operating characteristic curves of >0.9. Of these, TAT complexes and sTF correctly classified scrub typhus patients at the optimal cut-offs of ≥14.6 ng/mL and ≥387 pg/mL in 94.2% and 97.5% of the cohort, respectively, and VWF, sTM and tPA correctly classified murine typhus patients at the optimal cut-offs of ≥256%, ≥378 ng/mL and ≥14.5 ng/mL in 87.6%, 97.5% and 86.0% of the cohort, respectively (Table S1; Fig. S2).

### Response to treatment

After treatment with doxycycline (100 mg twice daily for 7 days following a 200-mg loading dose), the levels of all 15 coagulation and cytokine markers tended to return to those observed in healthy controls (Fig. S1; cytokine data not shown). Normalization of these parameters coincided with clinical improvement in response to antibiotic therapy.

## Discussion

To our knowledge, this is the first study to compare markers of coagulation and inflammation in sympatric scrub typhus and murine typhus patients in the same population. This study from Laos demonstrates that all markers of coagulation, fibrinolysis and endothelium activation are significantly affected in both typhus groups as compared with healthy controls, and that the coagulation profiles of scrub and murine typhus patients are profoundly different (Table 2).

Both typhus forms demonstrated similar disease severities and complication rates, but the levels of albumin (reduced, but within the normal range) and CRP (elevated) in patients with scrub typhus suggest that inflammation might play a more important role in scrub typhus than in murine typhus (Table 1). In spotted fever group disease, the most important pathophysiological effect of rickettsial infection is increased vascular permeability with extravascular loss of albumin. Whether this might play a role in scrub typhus as well remains to be investigated.

Coagulation changes in scrub typhus patients were characterized by an inflammation-induced coagulopathy, with coagulation activation, AT depletion, increases in the levels of proinflammatory cytokines and more pronounced CRP levels than in murine typhus patients. The level of tissue factor (TF), the main initiator of coagulation activation through the so-called extrinsic pathway, was so prominently raised in scrub typhus patients that it could be used to correctly classify 97.5% of all patients in this study (Table S1).

Proinflammatory cytokines such as TNF-α, IL-1β and IL-6 have been shown to induce TF expression on endothelial cells [14] and mononuclear cells upon activation [15]. The association of *O. tsutsugamushi* with circulating mononuclear cells *in vivo* [16], and previous findings of elevated soluble L-selectin levels in patients with scrub typhus, are both suggestive of mononuclear cell activation [9], and CRP, the level of which is more prominently increased in scrub typhus, has been shown to facilitate monocyte–endothelial cell interactions [17] and to promote PAI-1 and TF formation [18]. Despite the procoagulant profile of patients with scrub typhus, no overt DIC was observed in this study, although occasional case reports have described DIC in scrub typhus [19].

The activation pattern in murine typhus patients followed a distinct ‘endothelial perturbation profile’, with prominent increases in the levels of endothelium-derived factors, including VWF, sTM, tPA, and PAI-1. Thrombomodulin is an anticoagulant transmembrane glycoprotein and an endothelial marker, and plasma levels of its proteolytically degraded soluble form correlate with endothelial activation and organ damage [20]. The sTM level was so prominently raised in murine typhus patients, that, like sTF in scrub typhus patients, it could be used to correctly classify 97.5% of all patients in this study (Table S1).

Activated PC antagonizes TF-induced coagulation activation. PC is activated by thrombin when it is bound to thrombomodulin on the endothelial surface, and inhibits thrombin-mediated conversion of fibrinogen to fibrin, and binding of thrombin to other cellular receptors on platelets or inflammatory cells [21]. This anticoagulant PC system appeared to be less active in murine typhus patients, with significantly lower PC levels than in the other patient groups (Fig. 2). The high concentrations of endothelium-derived PAI-1 observed in murine typhus patients may explain the decrease in overall fibrinolytic activity measured as PAA despite the high concentrations of tPA in this group (Fig. 4).

The plasma levels of cytokines were generally low, which could be attributable to predominant local production at the site of inoculation eschar and/or disseminated small foci of infection with a dilution effect on the systemic compartment [22]. The low levels of TNF-α and IL-1β observed upon admission appear to be consistent with a previous study in Vietnam, where TNF-α and IL-1β levels in scrub typhus patients were undetectable in 90% and 75%, respectively [23]. IL-10 levels were four-fold higher in scrub typhus than in murine typhus patients, suggesting a stronger anti-inflammatory tendency with suppression of other inflammatory cytokines such as TNF-α, IL-1β, and IL-6 (Table 2), especially in bacteraemic patients with scrub typhus, who had significantly higher IL-8 and IL-10 levels and significantly lower PAA levels on admission, in contrast to murine typhus patients, in whom no association between bacteraemia and cytokine levels was noted. IL-10 can inhibit the expression of major histocompatibility complex class II antigens, CD54 (ICAM-1), CD80 (B7.1), and CD86 (B7.2), on monocytes, through a post-transcriptional mechanism. This could represent an immunomodulatory host–pathogen effect, as the T-cell-activating capacity of monocyte antigen presentation is reduced [24]. Furthermore, IL-10 can attenuate activation of the coagulation system, inhibit cytokine release, and potently modulate the fibrinolytic system, resulting in an inhibition of procoagulant responses in humans with induced endotoxaemia [25], thus raising further questions regarding its role in scrub typhus.

This study highlighted the diagnostic and highly discriminatory potential of these coagulation markers for scrub and murine typhus (all p <0.0001; Table 2), as they correctly classified patients with either form of typhus. sTF at a cut-off of ≥387 pg/mL had the strongest predictive power for scrub typhus, correctly classifying 97.5% of all patients (corresponding to a sensitivity of 98.2% and a specificity of 97%), and sTM scored highest for murine typhus, correctly classifying 97.5% of all patients (sensitivity of 96.4% and specificity of 98.5%) at the designated cut-off (Table S1; Fig. S2). These findings warrant further investigations into the diagnostic potential of these markers.

The endothelium-tropic spotted fever rickettsia *R. rickettsii* (Rocky Mountain spotted fever) and *R. conorii* (Mediterranean spotted fever) induce a procoagulant state, with upregulation of TF [26], downregulation of TM [27] and release of both PAI-1 [28] and VWF [29] in cultured human endothelial cells. These data support the profile seen in human plasma samples from murine typhus patients in this study, which is also in line with the evidence for the endothelial tropism of *R. typhi* derived from *in vitro* experiments, mouse studies, and human post-mortem studies [7,30]. However, the pattern seen in scrub typhus supports a procoagulant state with a less ‘endothelial’ but more inflammatory profile. This could suggest more prominent mononuclear involvement, which is supported by evidence of significantly higher mononuclear cell activation in scrub than in murine typhus [9]. Furthermore, dendritic and mononuclear cell infection with *O. tsutsugamushi* could have a role in the disease process beyond the early immune response, which may include a role in early systemic dissemination (and replication?) via circulation of these cell types beyond the skin inoculation sites.

In conclusion, this study shows activation of coagulation in both scrub and murine typhus patients as compared with controls. In scrub typhus, coagulation activation is more prominent and related to a stronger proinflammatory response, whereas in murine typhus, changes in coagulation and fibrinolysis follow a pattern suggestive of endothelial cell perturbation. Further studies are needed to improve our understanding of the pathophysiological changes underlying these disease states, in order to improve diagnosis and ultimately to help in the design of effective vaccines for both typhus diseases.

## Transparency Declaration

This work was supported by the Wellcome Trust of Great Britain as part of the Wellcome Trust–Mahidol University–Oxford Tropical Medicine Research Programme. D. H. Paris holds a Wellcome Trust Clinical Research Training Fellowship (Grant No. 078990/Z/06/Z). The funders had no role in the study design, data collection and analysis, decision to publish, or preparation of the manuscript. All authors declare that they do not have a commercial or other association that might pose a conflict of interest.

## Addendum

D. H. Paris, E. C. Löwenberg, A. M. Dondorp, and N. P. J. Day: wrote the paper. P. N. Newton, T. van der Poll, M. Levi, and N. P. J. Day: designed the study. D. H. Paris, V. Chansamouth, P. Nawtaisong, R. Phetsouvanh, S. D. Blacksell, and P. N. Newton: responsible for clinical and diagnostic data. D. H. Paris, E. C. Löwenberg, and P. Nawtaisong: performed laboratory experiments. D. H. Paris, S. J. Lee, T. van der Poll, M. Levi, and N. P. J. Day: analysed the data.

## Supporting Information

**Figure S1.** Changes in markers of coagulation, fibrinolysis and endothelium activation in response to therapy.

**Figure S2.** Receiver operating characteristic curves of the coagulation parameters with highest power of prediction for scrub typhus and for murine typhus.

**Table S1.** Coagulation parameters with high level of disease prediction.

## References

[1] Suttinont C, Losuwanaluk K, Niwatayakul K (2006). Causes of acute, undifferentiated, febrile illness in rural Thailand: results of a prospective observational study. Ann Trop Med Parasitol.

[2] Phongmany S, Rolain JM, Phetsouvanh R (2006). Rickettsial infections and fever, Vientiane, Laos. Emerg Infect Dis.

[3] Jensenius M, Fournier PE, Raoult D (2004). Rickettsioses and the international traveler. Clin Infect Dis.

[4] Blacksell SD, Bryant NJ, Paris DH, Doust JA, Sakoda Y, Day NP (2007). Scrub typhus serologic testing with the indirect immunofluorescence method as a diagnostic gold standard: a lack of consensus leads to a lot of confusion. Clin Infect Dis.

[5] Tsay RW, Chang FY (1998). Serious complications in scrub typhus. J Microbiol Immunol Infect.

[6] Walker DH, Harrison A, Henderson F, Murphy FA (1977). Identification of *Rickettsia rickettsii* in a guinea pig model by immunofluorescent and electron microscopic techniques. Am J Pathol.

[7] Walker DH, Feng HM, Ladner S (1997). Immunohistochemical diagnosis of typhus rickettsioses using an anti-lipopolysaccharide monoclonal antibody. Mod Pathol.

[8] Moron CG, Popov VL, Feng HM, Wear D, Walker DH (2001). Identification of the target cells of *Orientia tsutsugamushi* in human cases of scrub typhus. Mod Pathol.

[9] Paris DH, Jenjaroen K, Blacksell SD (2008). Differential patterns of endothelial and leucocyte activation in ‘typhus-like’ illnesses in Laos and Thailand. Clin Exp Immunol.

[10] Paris DH, Aukkanit N, Jenjaroen K, Blacksell SD, Day NP (2009). A highly sensitive quantitative real-time PCR assay based on the *groEL* gene of contemporary Thai strains of *Orientia tsutsugamushi*. Clin Microbiol Infect.

[11] Henry KM, Jiang J, Rozmajzl PJ, Azad AF, Macaluso KR, Richards AL (2007). Development of quantitative real-time PCR assays to detect *Rickettsia typhi* and *Rickettsia felis*, the causative agents of murine typhus and flea-borne spotted fever. Mol Cell Probes.

[12] Paris DH, Blacksell SD, Stenos J (2008). Real-time multiplex PCR assay for detection and differentiation of rickettsiae and orientiae. Trans R Soc Trop Med Hyg.

[13] Löwenberg EC, Charunwatthana P, Cohen S (2010). Severe malaria is associated with a deficiency of von Willebrand factor cleaving protease, ADAMTS13. Thromb Haemost.

[14] Levi M, van der Poll T, ten Cate H, van Deventer SJ (1997). The cytokine-mediated imbalance between coagulant and anticoagulant mechanisms in sepsis and endotoxaemia. Eur J Clin Invest.

[15] Osterud B, Rao LV, Olsen JO (2000). Induction of tissue factor expression in whole blood: lack of evidence for the presence of tissue factor expression in granulocytes. Thromb Haemost.

[16] Walsh DS, Myint KS, Kantipong P, Jongsakul K, Watt G (2001). *Orientia tsutsugamushi* in peripheral white blood cells of patients with acute scrub typhus. Am J Trop Med Hyg.

[17] Han KH, Hong KH, Park JH (2004). C-reactive protein promotes monocyte chemoattractant protein-1-mediated chemotaxis through upregulating CC chemokine receptor 2 expression in human monocytes. Circulation.

[18] Cermak J, Key NS, Bach RR, Balla J, Jacob HS, Vercellotti GM (1993). C-reactive protein induces human peripheral blood monocytes to synthesize tissue factor. Blood.

[19] Ben RJ, Feng NH, Ku CS (1999). Meningoencephalitis, myocarditis and disseminated intravascular coagulation in a patient with scrub typhus. J Microbiol Immunol Infect.

[20] Levi M, van der Poll T (2008). The role of natural anticoagulants in the pathogenesis and management of systemic activation of coagulation and inflammation in critically ill patients. Semin Thromb Hemost.

[21] Schouten M, Wiersinga WJ, Levi M, van der Poll T (2008). Inflammation, endothelium, and coagulation in sepsis. J Leukoc Biol.

[22] Cavaillon JM, Munoz C, Fitting C, Misset B, Carlet J (1992). Circulating cytokines: the tip of the iceberg?. Circ Shock.

[23] Kramme S, An le V, Khoa ND (2009). *Orientia tsutsugamushi* bacteremia and cytokine levels in Vietnamese scrub typhus patients. J Clin Microbiol.

[24] de Waal Malefyt R, Haanen J, Spits H (1991). Interleukin 10 (IL-10) and viral IL-10 strongly reduce antigen-specific human T cell proliferation by diminishing the antigen-presenting capacity of monocytes via downregulation of class II major histocompatibility complex expression. J Exp Med.

[25] Pajkrt D, van der Poll T, Levi M (1997). Interleukin-10 inhibits activation of coagulation and fibrinolysis during human endotoxemia. Blood.

[26] Sporn LA, Haidaris PJ, Shi RJ, Nemerson Y, Silverman DJ, Marder VJ (1994). *Rickettsia rickettsii* infection of cultured human endothelial cells induces tissue factor expression. Blood.

[27] Teysseire N, Arnoux D, George F, Sampol J, Raoult D (1992). von Willebrand factor release and thrombomodulin and tissue factor expression in *Rickettsia conorii*-infected endothelial cells. Infect Immun.

[28] Drancourt M, Alessi MC, Levy PY, Juhan-Vague I, Raoult D (1990). Secretion of tissue-type plasminogen activator and plasminogen activator inhibitor by *Rickettsia conorii*- and *Rickettsia rickettsii*-infected cultured endothelial cells. Infect Immun.

[29] Sporn LA, Shi RJ, Lawrence SO, Silverman DJ, Marder VJ (1991). *Rickettsia rickettsii* infection of cultured endothelial cells induces release of large von Willebrand factor multimers from Weibel–Palade bodies. Blood.

[30] Walker DH, Popov VL, Feng HM (2000). Establishment of a novel endothelial target mouse model of a typhus group rickettsiosis: evidence for critical roles for gamma interferon and CD8 T lymphocytes. Lab Invest.

