# Supplementary figures and images for: Coagulation and inflammation in scrub typhus and murine typhus—a prospective comparative study from Laos
Source: Clin Microbiol Infect. 2011 Nov 7;18(12):1221–8. doi: 10.1111/j.1469-0691.2011.03717.x (PMC3533763; doi:10.1111/j.1469-0691.2011.03717.x)

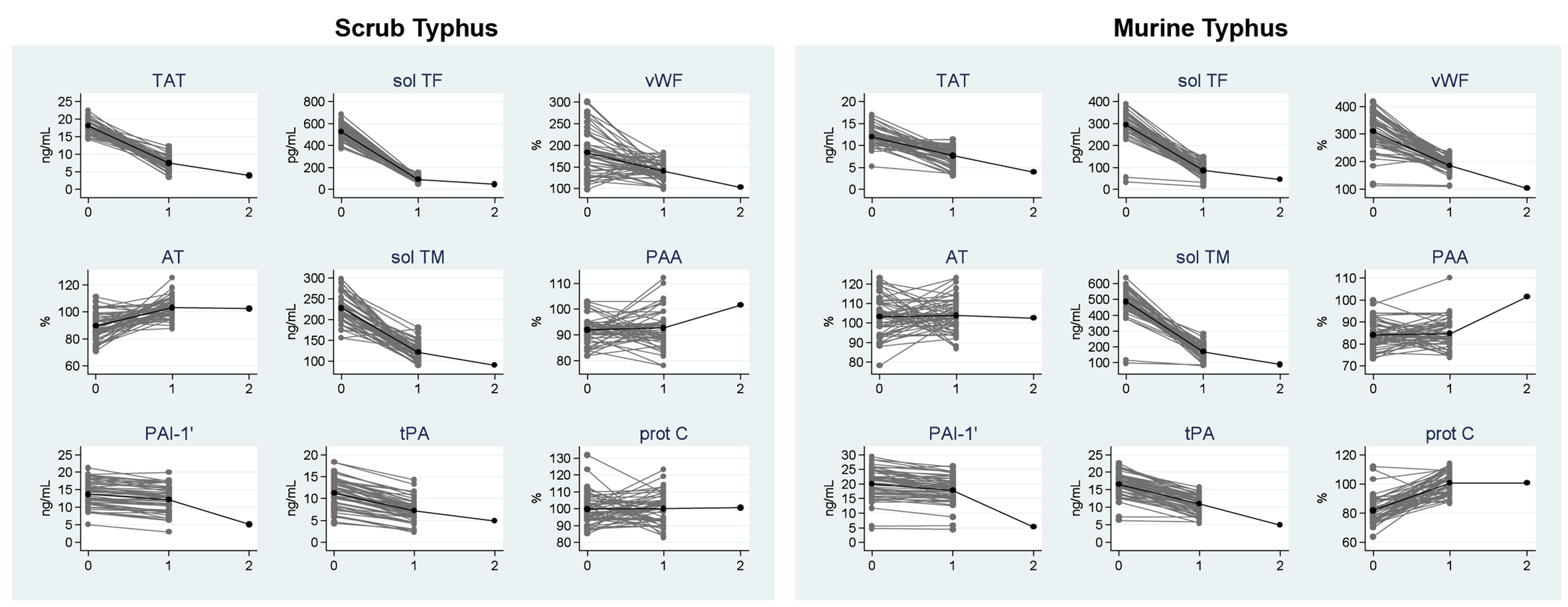

Supplement: Supplementary file 1 [file clm0018-1221-SD1.tif]

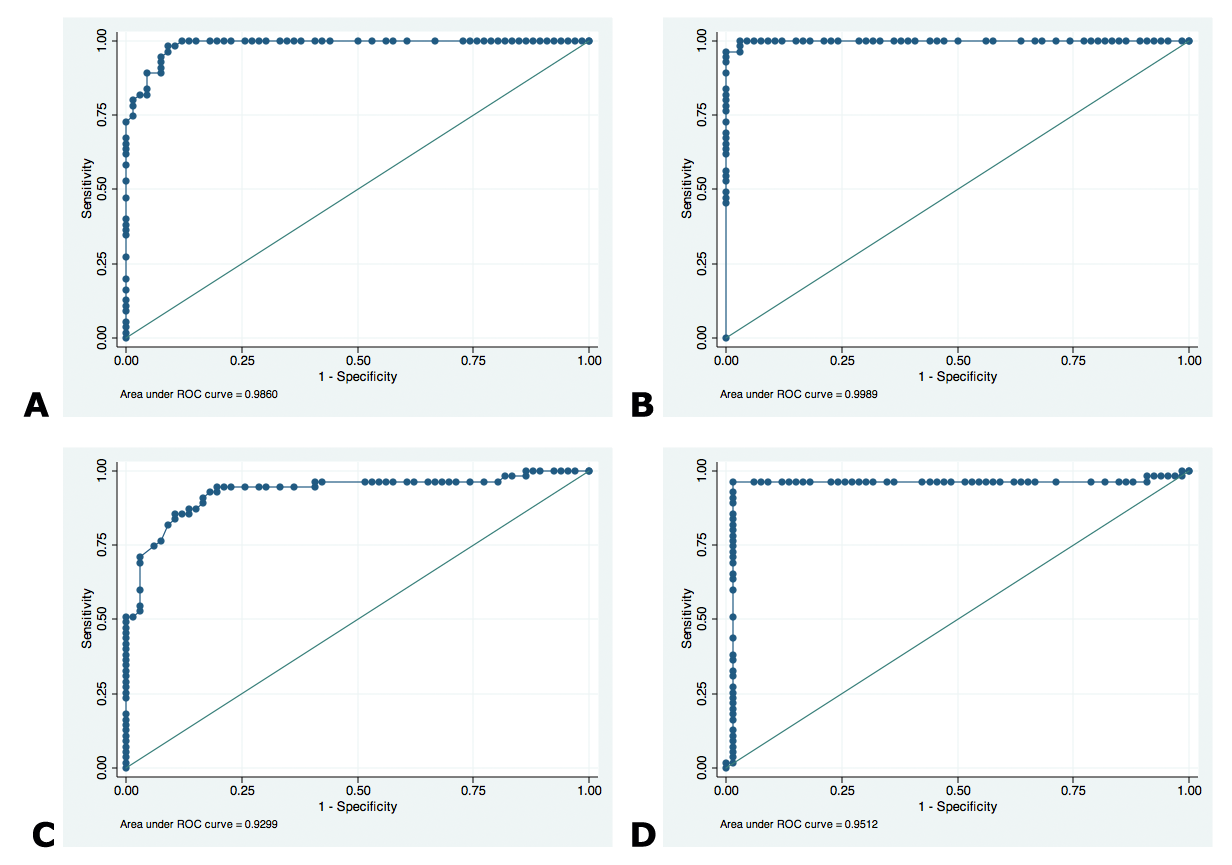

Supplement: Supplementary file 2 [file clm0018-1221-SD2.tif]
