# Supplementary material for: Coagulation and inflammation in scrub typhus and murine typhus—a prospective comparative study from Laos
Source: Clin Microbiol Infect. 2011 Nov 7;18(12):1221–8. doi: 10.1111/j.1469-0691.2011.03717.x (PMC3533763; doi:10.1111/j.1469-0691.2011.03717.x)
Supplement: Supplementary file 3 [file clm0018-1221-SD3.doc]

**Supporting Information**

**Table S1. Coagulation parameters with high level of disease prediction.**

| **Parameter** | **ROC area** | **95% CI** |  | **Cut-off** | **Unit** | **Youden’s Index** | **Sens** | **Spec** | **Correctly classified (%)** |
| --- | --- | --- | --- | --- | --- | --- | --- | --- | --- |
| **Scrub Typhus** | |  |  |  |  |  |  |  |  |
| TATc | 0.986 | 0.972 – 1.000 |  | ≥ 14.6 | ng/mL | 0.891 | 98.2 | 90.9 | 94.2 |
| sTF | 0.999 | 0.997 - 1.000 |  | ≥ 387 | pg/mL | 0.952 | 98.2 | 97.0 | 97.5 |
| **Murine Typhus** | |  |  |  |  |  |  |  |  |
| vWF | 0.930 | 0.880 - 0.980 |  | ≥ 256 | % | 0.749 | 85.5 | 89.4 | 87.6 |
| sTM | 0.951 | 0.897 - 1.000 |  | ≥ 378 | ng/mL | 0.948 | 96.4 | 98.5 | 97.5 |
| tPA | 0.905 | 0.849 - 0.961 |  | ≥ 14.5 | ng/mL | 0.712 | 81.8 | 89.4 | 86.0 |

**Footnote:** Coagulation factors with an area under ROC curve values >0.9 are included in this table. The cut-off value was chosen according to Youden's index (Y = Sensitivity+Specificity−1, which reflects the likelihood of a positive result among truly positive subjects versus that for negative subjects; it ranges from 0 to 1). The coagulation parameters with the highest percentage of correctly classified cases were sTF and sTM for scrub typhus and murine typhus respectively, when compared to the gold standard diagnosis.

Abbreviations: TATc: thrombin-antithrombin complexes; sTF: soluble tissue factor; sTM: soluble thrombomodulin; vWF: von Willebrand factor; tPA: tissue type plasminogen activator.
