# Supplementary material for: Coagulation and inflammation in scrub typhus and murine typhus—a prospective comparative study from Laos
Source: Clin Microbiol Infect. 2011 Nov 7;18(12):1221–8. doi: 10.1111/j.1469-0691.2011.03717.x (PMC3533763; doi:10.1111/j.1469-0691.2011.03717.x)
Supplement: Supplementary file 4 [file clm0018-1221-SD4.doc]

**Supplemental Figure legends:**

**Figure S1. Changes in markers of coagulation, fibrinolysis and endothelium activation in response to therapy.**

Between admission and follow-up, the levels of all factors tended to normalize to levels observed in healthy controls. The median [IQR] time from admission to follow up was 6 days [IQR 4-7] for both ST and MT. Paired patient samples are connected by the grey lines and medians by black lines, x-axis: admission = 0, follow up = 1, healthy control = 2.

**Figure S2. ROC curves of the coagulation parameters with highest power of prediction for scrub typhus and for murine typhus.**

Based on paired dynamic serology as gold standard diagnosis, the admission plasma levels of sTF correctly classified 97.5% of all patients presenting with scrub typhus (corresponding sensitivity 98.2% and specificity 97%) and similarly, sTM correctly predicted 97.5% of patients with murine typhus (sensitivity 96.4% and specificity 98.5%) at the designated cut-off (see also supplementary Table S1).
